# Individual-level socioeconomic status and community-level inequality as determinants of stigma towards persons living with HIV who inject drugs in Thai Nguyen, Vietnam

**DOI:** 10.7448/IAS.16.3.18637

**Published:** 2013-11-13

**Authors:** Travis Lim, Carla Zelaya, Carl Latkin, Vu Minh Quan, Constantine Frangakis, Tran Viet Ha, Nguyen Le Minh, Vivian Go

**Affiliations:** 1 Department of International Health, Johns Hopkins Bloomberg School of Public Health Baltimore, MD, USA; 2 Department of Epidemiology, Johns Hopkins Bloomberg School of Public Health Baltimore, MD, USA; 3 Department of Health, Behavior and Society, Johns Hopkins Bloomberg School of Public Health Baltimore, MD, USA; 4 Department of Biostatistics, Johns Hopkins Bloomberg School of Public Health Baltimore, MD, USA; 5 Thai Nguyen Center for Preventive Medicine, Thai Nguyen, Vietnam

**Keywords:** stigma, injection drug users, persons who inject drugs, HIV, income inequality, socioeconomic status, GINI coefficient, multi-level model, social determinants of health

## Abstract

**Introduction:**

HIV infection may be affected by multiple complex socioeconomic status (SES) factors, especially individual socioeconomic disadvantage and community-level inequality. At the same time, stigma towards HIV and marginalized groups has exacerbated persistent concentrated epidemics among key populations, such as persons who inject drugs (PWID) in Vietnam. Stigma researchers argue that stigma fundamentally depends on the existence of economic power differences in a community. In rapidly growing economies like Vietnam, the increasing gap in income and education levels, as well as an individual's absolute income and education, may create social conditions that facilitate stigma related to injecting drug use and HIV.

**Methods:**

A cross-sectional baseline survey assessing different types of stigma and key socioeconomic characteristics was administered to 1674 PWID and 1349 community members living in physical proximity throughout the 32 communes in Thai Nguyen province, Vietnam. We created four stigma scales, including HIV-related and drug-related stigma reported by both PWID and community members. We then used ecologic Spearman's correlation, ordinary least-squares regression and multi-level generalized estimating equations to examine community-level inequality associations, individual-level SES associations and multi-level SES associations with different types of stigma, respectively.

**Results:**

There was little urban–rural difference in stigma among communes. Higher income inequality was marginally associated with drug-related stigma reported by community members (*p*=0.087), and higher education inequality was significantly associated with higher HIV-related stigma reported by both PWID and community members (*p*<0.05). For individuals, higher education was significantly associated with lower stigma (HIV and drug related) reported by both PWID and community members. Part-time employed PWID reported more experiences and perceptions of drug-related stigma, while conversely unemployed community members reported enacting lower drug-related stigma. Multi-level analysis revealed that the relationship between education inequality and HIV-related stigma is superseded by the effect of individual-level education.

**Conclusions:**

The results of the study confirm that socioeconomic factors at both the individual level and community level affect different types of stigma in different ways. Attention should be paid to these differences when planning structural or educational interventions to reduce stigma, and additional research should investigate the mechanisms with which SES and inequality affect social relationships and, in turn, stigma.

## Introduction

Stigma towards persons living with HIV (PLHIV) and key populations at higher risk of HIV infection is a major barrier to curbing the HIV epidemic [1–4]. Research has shown that HIV-related and drug-related stigma can undermine HIV prevention efforts [5, 6] by negatively affecting HIV test-seeking behaviour [7–9], willingness to disclose HIV status, health-seeking behaviour [10, 11] and quality of healthcare received [12, 13]. Parker and Aggleton [14] described a conceptual framework of stigma, which was pivotal in highlighting the socioeconomic differences between groups as central conditions that may facilitate stigma through the reinforcement of differences, imbalance of power, and loss of social status [14]. This, and other related work [15, 16], emphasizes the need to need to intervene on stigma at a social level rather than at the level of individual emotional responses and beliefs concerning HIV and AIDS. However, there have been few studies to explore the hypothesized structural processes with quantitative statistical methods.

Because stigma is an inherently social phenomenon composed of the interactions between people, it follows that phenomena which disrupt interpersonal interaction could also reinforce stigma; socioeconomic inequalities are believed to have detrimental effects on social interactions, especially by reducing social capital and social trust [17–21]. The consequences of low social capital resulting from socioeconomic inequalities have characteristics remarkably similar to those of stigma, including social differentiation, prejudice and social exclusion. Indeed, several papers have in turn demonstrated that decreased social capital is associated with more expression and perception of HIV-related stigmatizing attitudes in the community [22, 23]. This suggests at least one pathway in which socioeconomic inequality (e.g., income or education inequality) perpetuates stigma indirectly through reduced social capital.

However, there is also evidence to support the idea that individuals’ social interactions are shaped by their own personal circumstances [24, 25]. At the individual level, expression of HIV-related stigma and discrimination among community members has been shown to be greater among those with less education in several low-income settings [26–29], partially due to a lack of understanding of modes of HIV transmission compared to the educated (i.e., who have less fear of infection through casual contact) [29]. Reported experience of stigma by PLHIV was more also pronounced among those who are poor [28, 30, 31], possibly because they have fewer resources available to conceal their HIV status and/or mitigate negative responses from society. Additionally, HIV-related stigma and discrimination in employment and housing reduce the stability and therefore the socioeconomic status (SES) of individuals living with HIV [32, 33], and these consequences may potentially be extended to family members who are stigmatized by association [34]. However, few studies have compared the association of both SES inequalities and individual SES and stigma in the same setting.

Research on the separate contributions of community-level and individual-level factors, such as social determinants of health within neighbourhoods, has received considerable attention [35–37] and may be applied to stigma research. Social determinants of health at both the community and individual levels are also known to be determinants of HIV infection, and therefore are candidate factors that might relate HIV infection and HIV stigma. For example, the socioeconomic status of an individual may affect HIV infection risk [38–41] and affect HIV disease prognosis [42, 43]. In the reverse causal direction, HIV and AIDS place a significant economic burden on infected individuals as well as their families, as caregiving imposes a significant opportunity cost in lost wages to households caring for PLHIV [44, 45], a consequence that might be alleviated, but not eliminated, by free antiretroviral therapy (ART) [46]. Moreover, inequality within a country or a community is significantly associated with HIV burden, even more so than the average wealth of that community [47–51].

Vietnam may be an instructive setting for its confluence of stigma and social inequality. The HIV epidemic in Vietnam has been concentrated among persons who inject drugs (PWID), who currently comprise between 53 and 65% of HIV infections in the country [52]. Stigmatization of PLHIV in Vietnam has resulted, in part, from state-initiated propaganda campaigns against “social evils” which encouraged the identification of drug users to the authorities [53], and implied that drug use and sex work were to blame for the HIV epidemic [54]. The result of such programs may lead to what has been called “layered stigma” or “double stigma” in the literature [55], a combination of both drug-related stigma and HIV-related stigma, which is potentially more detrimental than either alone [56].

There have also been great economic changes in Vietnam over the past 25 years. The relative levels of income and education in Vietnam have diverged in response to “Doi Moi” economic policies which started in 1986 to gradually encourage more private enterprise, opening of markets, and increased industrialization, trade and investment [57]. This period of rapid economic growth has contributed to a shift from formerly socialist agricultural collectives towards more unequal wealth distribution, and the rise in non-agriculture wages is attributed to widening income gaps [58, 59] and a parallel increase in private schooling and tutoring.

Using the conceptual framework of Parker and Aggleton [14], and utilizing the measurement framework outlined by Stangl *et al*. [60], we will test the association between intersecting HIV- and drug-related stigma, and the socioeconomic inequalities that may drive, and be reinforced by, stigma. This will be done on two levels: firstly, we aim to determine what types of stigma may be associated with unequal distribution of socioeconomic resources at the community level; and, secondly, we aim to determine what types of stigma are associated with individual-level socioeconomic characteristics irrespective of the level of others. Given the literature on inequality, SES and HIV stigma, we hypothesize that in communities with higher socioeconomic inequalities the majority of community members will express more stigmatizing attitudes, while better educated individuals will hold fewer stigmatizing attitudes. We also hypothesize that PLHIV in communities with higher inequalities, and poorer and less educated PLHIV, will on average perceive higher levels of stigma. Finally, we will use multi-level regression analysis to determine if community-level income inequality mediates or modifies the associations of individual-level SES characteristics with stigma.

## Methods

### Study design and population

Cross-sectional data for this study were collected in Vietnam from the baseline visit of our study entitled “Prevention with positives: a randomized controlled trial among HIV-infected IDU,” a four-arm factorial design intervention that included both individual-level and community-level stigma reduction components. Briefly, 1674 male PWID, of whom 31% were living with HIV, and 1349 community members (40% male) were recruited from Thai Nguyen province for enrolment. PWID were recruited by active recruiters and peer referral; community members (who were not known to be injection drug users) were systematically sampled from the first consenting eligible adult living at the fifth house on the right from the PWID household. The community members and PWID were not revealed to one another at any point during the study. Additionally, of the PWID living with HIV (31%), the majority did not know their HIV status at the time they completed the baseline assessment (73%). After completion of the baseline assessment, all PWID were offered pre- and post-test counselling and two parallel rapid HIV tests (Determine: Abbott Laboratories, Abbott Park, IL; and Bioline: SD, Toronto, Canada), with same-day result return.

### Stigma measures

Both PWID and community members were asked to self-report on the following types of stigma:

1. *HIV-related stigma*. Both PWID and community members were asked to report on their (a) expression of shame, blame and social isolation towards PLHIV, (b) perceptions of HIV-related stigma and discrimination in the community and (c) support for equitable policies (i.e., two scales, with three domains each).
2. *Drug-related stigma*. In one scale, PWID were asked to report on their (a) experiences of stigma and discrimination, (b) internalized or self-stigma and (c) perceived stigma in the community (three domains). Conversely, in a separate scale, community members were asked to report on their perceptions of devaluation of IDU in their community (one domain).

We chose to divide HIV-related stigma items and drug-related stigma items *a priori*. All HIV-related stigma questions were adapted from stigma scales previously used and validated in other settings [54, 61–63]. Previously validated drug-related stigma questions did not exist from previous studies and were newly developed for this study.

The individual stigma scales (a set of four) used in regression analysis were calculated as the sum of scores ranging from one to four on a Likert scale of participants’ responses to statements assessing their opinions and attitudes towards HIV and drug use. A higher value is associated with more stigma, as positively phrased items were reverse coded. In addition, the means of individual item scores for each type of stigma were also calculated to facilitate comparisons between scales. We conducted an exploratory factor analysis (EFA) on each scale, removing items with uniqueness greater than 0.75. Scale reliability before and after item reduction was measured by calculating Cronbach's alpha (α). The highest reliability was sought by calculating the α if individual items were deleted (α_d_), thereby assessing the contribution of each item to the scale's reliability. Items that lowered a scale's overall reliability were removed, and final items are in the Supplementary file.

### Socioeconomic and inequality measures

Community members were asked about their average monthly incomes from all jobs and businesses, and also their incomes from supplemental sources such as government assistance and pensions. The amounts were summed to obtain total average monthly income. The study catchment province of Thai Nguyen was divided by its 32 administrative communes, which we used to define the unit of “community” in our study as they typically contained their own health centre and economic centre, and they were identifiable as either predominantly urban or rural. For each commune, Lorenz curves were plotted from the self-reported total incomes (employment and non-employment income) of community study participants. PWID incomes were not included, as they may not have been representative of the broader community. The GINI coefficient, a standard index for measuring inequality that falls between 0 and 1, was calculated for each commune from the Lorenz curves as described here [64]. We also created a GINI index for education by calculating the inequality in total years of education by commune. To account for possible GINI coefficient bias due to varying sample sizes from the different communes, GINI coefficients were normalized using a first-order correction factor of N/N-1 [65].

### Community-level analysis

For each commune, the mean stigma score for each of the four types of stigma measured was calculated. Correlation (both Spearman's correlation for sparse data and a sample-size weighted Pearson's correlation) was calculated between mean commune stigma and commune inequality. Due to the sample size of 32 communes within Thai Nguyen province for this analysis, as limited by the design of the parent study, we set a significance level of *p*<0.1 as our threshold of interest.

### Individual-level analysis

We examined total monthly income, level of education and employment status as self-reported in the questionnaire as predictors of stigma. For mean HIV-related and drug-related stigma reported by PWID, we used the PWID income, education and employment as the individual-level predictors. For HIV-related and drug-related stigma reported by community members, we used their income, education and employment.

### Ethics approval

The study was approved by the Johns Hopkins Bloomberg School of Public Health Institutional Review Board and the Thai Nguyen Center for Preventive Medicine Institutional Review Board.

### Regression and multi-level regressions

For both ordinary least squares (OLS) regression and multi-level generalized estimating equation (GEE) models, the outcome of stigma was modelled as a continuous scale variable composed of either drug-related stigma factors or HIV-related stigma factors. For the multi-level model of PWID, we treated PWID as clustered in networks nested in communes. For community members, participants were clustered in communes. Independent variables were socioeconomic factors at the individual and/or community level.

## Results

The socioeconomic indicators for the entire province of Thai Nguyen are summarized in Table 1. There was a wide range of monthly incomes (coefficient of variation = 0.847) across the study population, which translates into a GINI coefficient of 0.42, considerably higher than the average provincial estimate of 0.32 [66] and slightly above the national estimate of 0.38 [67]. Years of education had less variability because it has a finite range of values, and the overall GINI for education was 0.19. Education in Vietnam is relatively high: more than 93% of community members had higher than primary school education, including more than 90% of PWID.

**Table 1:** Province-wide estimates of commune characteristics and average stigma, not accounting for commune or other network clustering

	Overall (SD) or [SE]	Range	Urban (SD) or [SE]	Rural (SD) or [SE]
GINI coefficient income inequality	0.420 [0.009]	0.278–0.499 (communes)	0.407 [0.013]	0.431 [0.012]
GINI coefficient educational attainment	0.194 [0.003]	0.125–0.276 (communes)	0.173 [0.004]	0.189 [0.005]
Median income, USD	$92.59 (78.45)	$0.5–$588.24	97.93 (81.53)	87.45 (75.06)
Median years of education	9.67 (3.37)	0–18	10.84 (3.33)	8.57 (3.00)
Summary of four stigma scales – main outcomes of interest				
Total HIV-related stigma reported by PWID	40.35 (3.32)	21–52	40.16 (3.44)	40.61 (3.14)
Average response for HIV-related stigma items (PWID)	2.37	1–4	2.36	2.39
Total drug-related stigma reported by PWID	15.88 (2.64)	6–24	15.93 (2.60)	15.82 (2.69)
Average response for drug-related stigma items (PWID)	2.65	1–4	2.66	2.64
Total HIV-related stigma, reported by community (non-PWID)	38.09 (5.10)	18–56	37.50 (5.04)	38.67 (5.09)
Average response for HIV-related stigma items (non-PWID)	2.12	1–4	1.97	2.04
Total drug-related stigma reported by community (non-PWID)	10.88 (1.48)	7–16	10.83 (1.57)	10.92 (1.39)
Average response for drug-related stigma items (non-PWID)	2.72	1–4	2.71	2.73

The drug-related stigma domain had fewer valid unique items, and therefore its scales were generally shorter compared to the HIV-related stigma scales.

### HIV-related stigma measures

For PWID, 17 items comprised the final HIV-related stigma scale (Cronbach's alpha = 0.85). If PWID had all “agreed” about each HIV-related Likert scale stigma item, the mean score would be 3 on a 4-point scale, and if they had all “disagreed” about the HIV stigma items, the mean score would be 2. In our sample of PWID, the mean score for HIV-related stigma, assuming equal weight for each item, was 2.37 (Table 1). For community members, 19 survey items comprised the final HIV-related stigma scale (Cronbach's alpha = 0.89). The mean score was 2.12, suggesting low expression and perception of HIV-related stigma reported by community members. Among both PWID and community members, all items loaded onto their three respective domains outlined *a priori*.

### Drug-related stigma measures

For PWID, six survey items were sufficiently unique to contribute to the total drug-related stigma scale (Cronbach's alpha = 0.81). The mean score for drug-related stigma was 2.65 on the 4-point scale. These items correctly loaded on two of the *a priori* domains of experienced and perceived stigma. All items belonging to the domain of internalized or self-stigma were dropped, as they did not load sufficiently in the EFA. For community members, four of the original five items were retained and comprised the final drug-related stigma scale (Cronbach's alpha = 0.72), with a mean score of 2.72.

Participants were recruited equally from urban and rural communes, with 57.8% of PWID and 48.7% of community participants living in predominantly urban communes of Thai Nguyen. Urban–rural differences encompass a subset of related socioeconomic and demographic factors; thus, we stratified communes by urban or rural based on their administrative designation (Table 1). As expected, both individual income and years of education were higher in urban communities. Surprisingly, income inequality and educational inequality were slightly higher in rural settings. However, none of the stigma scales were appreciably different when comparing urban to rural communes.

### Community-level socioeconomic inequality

We used adjusted GINI indices to look at the correlation between the four stigma scales and community-level distribution of income and years of education. Table 2 shows that income inequality is not significantly correlated with total HIV stigma reported by either PWID or community members at the community level, although communes with higher income inequality were correlated with higher drug-related stigma towards PWID (weighted correlation coefficient 0.33) with marginal statistical significance (*p*<0.1 level). Education inequality, estimated using the adjusted GINI coefficient for the community-level distribution of total years of education, was significantly correlated with both total HIV-related stigma reported by PWID and total HIV-related stigma reported by community members (*p*<0.05 level), but not with drug-related stigma scales (Table 3).

**Table 2:** Ecologic correlations between mean stigma scale and inequality, comparing four types of inequality by both income inequality (adjusted income GINI coefficient, top) and educational inequality (adjusted years of education GINI coefficient, bottom); *n*=32 communes

	Spearman's correlation coefficient	Pearson's
Income inequality and stigma		
Effect on total stigma reported by PWID		
HIV-related stigma	−0.1393	−0.0776
Drug-related stigma	0.1375	0.1620
Effect on total stigma reported by community		
HIV-related stigma	−0.1910	−0.1223
Drug-related stigma	0.2398	0.3316*
Commune-level educational inequality and stigma		
Effect on total stigma reported by PWID		
HIV-related stigma	0.3893**	0.3284*
Drug-related stigma	0.0183	0.0676
Effect on total stigma reported by community		
HIV-related stigma	0.4117**	0.4291**
Drug-related stigma	0.1617	0.0920

* Significant at the *p*<0.1 level.

** Significant at the *p*<0.05 level.

**Table 3:** Bivariate (unadjusted) associations between individual-level SES and individual-level stigma

	Effect on total stigma reported by PWID		Effect on total stigma reported by community (non-PWID)	
		
	HIV-related stigma	Drug-related stigma	HIV-related stigma	Drug-related stigma
Total average monthly income, USD	−0.000286 (0.00111)	−0.000676 (0.000885)	0.000241 (0.00183)	−0.000108 (0.000527)
Highest level of education completed				
Primary (reference)	–	–	–	–
Some secondary	−0.572 (0.280)**	−0.595 (0.223)***	−1.982 (0.568)***	−0.304 (0.163)*
Graduated high school	−1.328 (0.289)***	−0.392 (0.230)*	−3.662 (0.599)***	−0.515 (0.172)***
College or higher	−1.650 (0.396)***	−0.860 (0.316)***	−4.696 (0.614)***	−0.515 (0.177)***
Employment status				
Full-time (reference)	–	–		
Part-time	−0.376 (0.224)*	0.481 (0.176)***	−0.677 (0.544)	0.0865 (0.153)
Unemployed/retired/student	−0.316 (0.278)	0.0676 (0.220)	−0.341 (0.371)	−0.216 (0.106)**

Independent variables are characteristics of PWID (Column 2) or of non-PWID community members (Column 3). Each stigma coefficient is a separate simple OLS linear regression with a single predictor from the same individual reporting the stigma.

* Significant at the *p*<0.1 level.

** Significant at the *p*<0.05 level.

*** Significant at the *p*<0.01 level.

### Individual-level SES

We next examined the associations between our stigma scales and individual-level SES variables. Using bivariate OLS regression, we modelled the four stigma scales on monthly income, education level and employment status of the individual respondent who was reporting the stigma (Table 3). For bivariate associations, we wanted to ignore group-level effects, and thus we did not account for clustering at the commune level or the network level.

Compared to primary school education, having any high school or higher education was significantly associated (*p*<0.001) with lower stigma scores, in stigma of all types, and the effect was approximately dose dependent. Most PWID were employed either full-time (73.9%) or part-time (16.4%). Compared to PWID with full-time jobs, PWID with part-time jobs experienced significantly higher drug-related stigma (*p*=0.006). Unexpectedly, they also reported experiencing lower HIV-related stigma with marginal statistical significance (*p*=0.093).

To simultaneously account for the effects of individual-level SES and community-level inequality in SES, we created a full multi-level multivariate regression model using GEE to adjust for clustering at the commune level and the PWID network level. Four types of stigma (HIV-related reported by PWID, drug-related reported by PWID, HIV-related reported by the community and drug-related reported by the community) were modelled as outcomes as a function of both individual-level SES predictors and community-level inequality predictors used in the previous bivariate models.

The inclusion of both levels of SES in the model generally rendered community-level predictors statistically insignificant. The exception is that PWID in communes with higher median income reported perceiving significantly higher levels of drug-related stigma (*p*<0.05), and higher income inequality was associated with higher drug-related stigma enacted by community members, with marginal statistical significance (*p*<0.1); however, this finding was not found to be highly robust to different covariate combinations.

In the multi-level model, individual-level educational attainment remained associated with reduced stigma of all four categories, with high statistical significance. This individual-level education effect appears to negate the community-level effect of inequality in education, which had no statistically significant relationship in the full multi-level model. As with the bivariate case, PWID employed part-time reported higher total drug-related stigma compared to PWID employed full-time (Table 4).

**Table 4:** Full adjusted multi-level GEE model of stigma on individual-level and community-level covariates, accounting for clustering by district for non-PWID community members; coefficients are population average estimates

	Model	[1] Total HIV-related stigma reported by PWID (SE)	[2] Total drug-related stigma reported by PWID (SE)	[3] Total HIV-related stigma reported by community (SE)	[4] Total drug-related stigma reported by community (SE)
Individual-level factors	Highest level of education completed				
	Primary (reference)				
	Some secondary	−0.527 (0.282)*	−0.652 (0.224)***	−1.972 (0.559)***	−0.290 (0.165)*
	Graduated high school	−1.151 (0.296)***	−0.415 (0.235)*	−3.433 (0.608)***	−0.427 (0.180)**
	College or higher	−1.513 (0.405)***	−0.932 (0.324)***	−4.182 (0.651)***	−0.361 (0.194)*
	Employment status				
	Full-time (reference)	–	–	–	–
	Part-time	−0.315 (0.228)	0.494 (0.181)***	−0.326 (0.529)	0.185 (0.157)
	Unemployed/retired/ student	−0.0332 (0.280)	0.105 (0.222)	0.488 (0.381)	−0.223 (0.114)*
	Average total monthly income (USD)	0.000721 (0.00113)	−0.000225 (0.0009)	0.00295 (0.00188)	−0.000387 (0.000565)
	Age (in years)	0.00360 (0.0111)	0.0144 (0.00885)	0.0719 (0.0124)***	0.0130 (0.00371)***
Community	GINI coefficient, income	−1.270 (1.842)	0.177 (1.864)	−0.555 (2.843)	1.443 (0.842)*
	GINI coefficient, education	3.536 (4.156)	2.905 (4.143)	6.036 (6.576)	−0.577 (1.934)
	Urban (vs. rural)	−0.135 (0.250)	0.299 (0.261)	−0.540 (0.304)*	−0.0598 (0.0900)
	HIV prevalence	0.424 (1.101)	1.803 (1.124)	−2.538 (1.54)*	−0.541 (0.459)
	Median commune income	−0.00552 (0.00520)	0.00982 (0.00530)**	0.0001 (0.0090)	0.00200 (0.00265)

For PWID, a mixed-effects model accounts for clustering by injection networks nested within the district.

* Significant at the *p*<0.1 level.

** Significant at the *p*<0.05 level.

*** Significant at the *p*<0.01 level.

Finally, we added cross-level interaction terms between community-level and individual-level predictors into the multi-level GEE model. The previous models 1–4 in Table 3 assume that the effect of community-level inequality is the same regardless of individual-level SES. Adding cross-level interaction terms to each model allows for the effect of community-level inequality to vary depending on the individual's SES. To select the interaction terms, we checked significant or marginally significant predictors of stigma from other tables, especially Table 3. The effect of income inequality on enacted drug-related stigma appears to primarily effect community members who are employed part-time, rather than employed full-time or unemployed (*p*=0.087). However, the other cross-level effects were not statistically significant, indicating that the effect of SES inequality in the community did not vary by individual-level SES.

## Discussion

We have modelled the association between socioeconomic factors and four types of stigma: HIV-related stigma reported by PWID, drug-related stigma reported by PWID, HIV-related stigma reported by community members and drug-related stigma reported by community members. In this setting, reports of drug-related stigma were slightly higher than those of HIV-related stigma, according to both PWID and community members, who on average had higher endorsement of drug-related stigma items than for HIV-related stigma items. The findings in this study suggest that there is not a single dimension to stigma in Vietnam, but rather that each type of stigma has unique associations with individual-level SES and/or community-level SES inequality. Consequently, addressing socioeconomic factors may not uniformly lead to a reduction in each type of stigma. Public health interventions should take these differences into account to use appropriate strategies depending on the target population, type of stigma and community context.

Although urban and rural participants in our study differed significantly by socioeconomic characteristics, we found no urban–rural differences by any type of drug-related or HIV-related stigma. In the literature, urban–rural differences are significant predictors of HIV-related stigma in high-income countries [68, 69], with one study showing no urban–rural differences in low- and middle-income countries [70]. However, the distinction between urban and rural in Thai Nguyen may not have been as sharp or as updated as the administrative commune boundaries indicated.

At the community level, education inequality was correlated with HIV-related stigma reported by both PWID and community members. Income inequality is positively correlated with drug-related stigma reported by community members, but the statistical significance was marginal; the results may have been limited by commune sample size, or the effect of inequality may be distal to our observation of stigma, and obscured by proximal factors. In the context of Vietnam, income inequality may lead to a more judgmental attitude towards injecting drug users, who are perceived as not meeting expectations as providers of families [2, 71]. However, our results do not unequivocally confirm the prevailing stigma frameworks, which emphasize the central role of economic inequality [14–16].

At the individual level, drug-related stigma reported by PWID was associated with employment. PWID employed part-time reported higher drug-related stigma compared to those with full-time employment. Part-time employment was often reported as odd jobs, and the transient nature of this type of work may have reduced the social connections of these PWID with their community. Since underemployed PWID may not be able to fulfil their responsibility to provide for the family, a central tenet of Vietnamese society, PWID may have higher perceived stigma if they feel shame and pressure from failure to do so [72]. Our results suggest that employment interventions may help to counter drug-related stigma, possibly including combinations of community-level efforts like non-discrimination or privacy policies, plus individual-level efforts to educate employers about stigma, develop employable skills for PWID and/or re-integrate PWID into full-time employment. In other contexts, employment is a critical facilitator for re-integration after rehabilitation or detainment [73, 74]. We also found a complementary result: that individual-level unemployment among community members who were not PWID was associated with lower drug-related stigma, compared to employed community members. This may be, in part, because unemployed community members are less judgmental of PWID who are struggling like themselves.

As proposed *a priori*, higher education at the individual level was significantly associated with a reduction in all forms of stigma across all study participants. General education may be a proxy for a variety of factors, such as greater life experience, greater exposure to diversity or a higher level of HIV-specific knowledge. Knowledge about HIV has been shown to be associated with lower stigma due to greater understanding about transmission and risk (reviewed in Refs. [30, 75]). Given the association between education inequality and HIV-related stigma, it will be important to ensure that community members and PWID across various levels of education are reached with anti-stigma messaging tailored to the appropriate educational level.

Multi-level analysis, controlling for both individual-level and community-level factors together, did not markedly change the findings of the previous models. The notable exception was community-level education inequality, which lost its ecologic association with HIV-related stigma, a result which emphasizes that improving individual education may supersede the challenges of community inequality in education. Taken together, these findings suggest that interventions to reduce stigma would benefit most if they contain both individual-level and community-level components. Socioeconomic characteristics of communities could also give some strong indications on which areas would have the greatest need of such structural interventions (short of an actual stigma survey in each community).

### Limitations

It is possible that the relatively small number of communes within our sample made our analysis underpowered to detect the relationship between community-level income inequality and increased stigma. The results should be confirmed in other contexts using larger numbers of communities. In addition, it would be informative to study stigma among specific types of community members who may interact with PLHIV or PWID, such as employers or healthcare providers, who often influence social inequalities in the community broadly and towards PWID specifically. Future studies may collect more detailed income information or look at other measures of wealth such as expenditures or household assets, and could also collect primary data on social cohesion or social capital.

Previously validated drug-related stigma questionnaire items did not exist and were developed *de novo* for the parent study; furthermore, although the HIV-related stigma items were validated in other settings, they were not necessarily intended to be collapsed or combined. However, we found the newly compiled scales to be valid (adhering to *a priori* domains) and reliable in this study population. Responses to the stigma scale from community members may suffer from social desirability bias. If government mass communications to reduce stigma have been successfully disseminated in this area, respondents may have felt that it was important to respond in concordance with this government message, which would flatten the differences between reported stigma both within and between communities.

Finally, since this was a cross-sectional baseline survey, the directionality of the relationship between individual-level SES and stigma cannot be ascertained, especially for PWID whose SES may be directly affected by discrimination which in turn may affect their outlook, attitudes and coping mechanisms.

### Strengths

By studying both PWID and community members (not known to be PWID), we were able to examine the effect of community context and derive measures of inequality and wealth from one source – the broader community – and examine the effect on another, PWID. We were also able to examine two types of stigma (HIV and drug related), from the perspectives of both the source and the target of potential stigma. To our knowledge, this is also the first study that examines the socioeconomic determinants of stigma on multiple levels and their cross-level interactions. The method of sampling community members from their proximity to PWID households was a strength in that we intended to capture and measure a community microenvironment to increase the likelihood that PWID and community members are aware of one another and are affected by the same community-level context. However, our results are less generalizable to larger geographic settings, where PWID and other community members are less likely to encounter one another.

## Conclusions

Prevailing conceptual frameworks about the drivers of stigma posit that it causes, and is potentially facilitated by, inequalities between groups. Our findings on the relationship between stigma and inequality indicate that while inequalities are associated with stigma, individual-level factors such as education and employment can supersede the effects of inequality. Thus, even if broader social inequalities are complex and challenging to eliminate overall, specific interventions and policies that facilitate PWID employment and fill gaps in education and knowledge should make a tangible impact on stigma, and should be pursued by policy makers and practitioners. Given the rapid pace of economic development in Vietnam, it is important to detect negative social consequences such as increased stigma, and to ensure that neither HIV burden nor stigma is disproportionately affecting persons in lower social or economic strata.
